# Transition Cow Nutrition and Management Strategies of Dairy Herds in the Northeastern United States: Associations of Nutritional Strategies with Analytes, Health, Milk Yield, and Reproduction

**DOI:** 10.3390/ani13172701

**Published:** 2023-08-24

**Authors:** Allison L. Kerwin, Winfield S. Burhans, Daryl V. Nydam, Thomas R. Overton

**Affiliations:** 1 Department of Animal Science, Cornell University, Ithaca, NY 14853, USA; tro2@cornell.edu; 2 Dairy-Tech Group, South Albany, VT 05875, USA; dr.buzz@dtg-dairynutrition.com; 3 Department of Population Medicine and Diagnostic Sciences, College of Veterinary Medicine, Cornell University, Ithaca, NY 14853, USA; dvn2@cornell.edu

**Keywords:** transition cow, nutrition, performance, health, analytes

## Abstract

**Simple Summary:**

Evaluations of transition cow nutrition strategies on health and performance in larger commercial farms are limited. In a 72-farm prospective cohort study, we evaluated the associations of common nutritional strategies fed during the far-off dry, close-up dry, and fresh periods with postpartum health and performance. Overall, our results support feeding a controlled energy diet prepartum and high-starch fresh diet to primiparous and multiparous cows.

**Abstract:**

The objective was to identify relationships between transition cow nutritional strategies and the prevalence of elevated analytes (nonesterified fatty acids (NEFA), β-hydroxybutyrate (BHB), and haptoglobin (Hp)), disorder incidence (DI), milk yield, and reproductive performance. Multiparous and primiparous cows from 72 farms in the northeastern US were enrolled in a herd-level cohort study. Farms were dichotomized within parity into a nutritional strategy within each period; far-off: controlled energy (CE; <16.5% starch and ≥40% forage neutral detergent fiber (FNDF)) or not CE (NCE; ≥16.5% starch or <40% FNDF or both), close-up: high FNDF (HF; ≥40% FNDF) or low FNDF (LF; <40% FNDF), and fresh: low starch (LS; <25.5% starch) or high starch (HS; ≥25.5% starch). No evidence existed that transition cow nutritional strategies were associated with milk yield outcomes (*p* ≥ 0.20). In general, our results support feeding multiparous cows HF close-up and HS fresh to minimize excessive BHB and DI; however, multiparous cows fed LF close-up had a higher pregnancy rate, and lower prepartum NEFA and Hp. Similarly, our results support feeding primiparous cows CE far-off, HF close-up, and HS fresh to maximize reproductive performance, and minimize BHB and DI; however, herds fed HF close-up or HS fresh had higher Hp.

## 1. Introduction

Nutrition strategy recommendations during the transition cow period are often driven by results from controlled research trials or anecdotal observations. Although research exists evaluating transition cow nutritional strategies, large-scale data availability is limited, particularly for the periparturient and fresh cow periods. In addition, controlled research trials are often completed in tiestall barns, removing many influences of environment and management, potentially resulting in varying outcomes in freestall herds.

Various transition cow nutritional strategies have been investigated and recommended over the years [1,2,3]. The adoption of a controlled energy diet throughout the dry period has increased amongst the dairy industry and has been supported by controlled research trials for improving postpartum health [4,5,6]; however, one study has demonstrated decreased milk yield in cows fed a controlled energy prepartum diet [7]. In addition, it is still common to observe farms feeding a high energy far-off dry diet or a “steam-up” diet where the energy concentration of the diet is increased during the close-up period, even though previous studies have reported negative postpartum outcomes [5,8,9]. The hepatic oxidation theory proposes limiting fermentable starch intake and supply energy to meet energy requirements during the fresh period in order to improve milk yield compared to oversupplying fermentable starch and energy during the fresh period [10]; however, data are lacking or have not fully supported this theory [11,12,13].

The objectives were to identify observational relationships between dry-period (far-off and close-up strategies) and periparturient (close-up and fresh strategies)-period nutritional strategies, as characterized by dietary contents of starch, forage neutral detergent fiber (NDF), or both, and metabolic- (nonesterified fatty acids (NEFA) and β-hydroxybutyrate (BHB)) and inflammation-related (haptoglobin (Hp)) analytes, health disorders, milk yield, and reproductive performance. We hypothesized that: (1) controlled energy far-off and high-forage NDF close-up-fed herds would have lower prevalence of elevated postpartum biomarkers, lower disorder incidence, no difference in milk yield, and improved reproductive performance than herds fed a controlled or not-controlled energy far-off with a low-forage NDF close-up diet, (2) high-forage NDF close-up-fed herds will produce more milk but have a higher prevalence of elevated Hp when transitioning to a high-starch fresh diet versus a low-starch fresh diet, and (3) high-starch fresh-fed herds will produce more milk, have a lower prevalence of elevated NEFA and BHB concentrations but higher prevalence of elevated Hp concentrations, and have lower disorder incidence than low-starch fresh-fed herds. In addition to our specified hypotheses, all nutrition strategy comparisons were assessed to explore observational relationships.

## 2. Materials and Methods

### 2.1. Study Population and Study Design

A more complete description of the study design and study population was provided previously by Kerwin et al. [14]. In brief, a prospective cohort study was conducted from a convenience sample of 72 farms located in New York and Vermont between November 2012 and August 2015. All procedures involving cows in this study were approved by the Cornell University Institutional Animal Care and Use Committee, protocol # 2012-0124. Inclusion criteria for farms were: (1) Holstein herds, (2) ≥400 milking cows, (3) freestall housing, (4) total mixed ration (TMR)-fed herds, and (5) enrolled in monthly Dairy Herd Information Association (DHIA) testing or have on-farm milk recording with record management by Dairy Comp 305 (Dairy Comp 305, Valley Ag Software, Tulare, CA, USA) or PCDART (PCDART, Dairy Records Management System, Raleigh, NC, USA).

Farms were visited 3 times and characterization and sampling was focused on the same cohort of cows during the far-off dry (from 49 to 28 d prior to expected parturition), close-up dry (from 21 to 0 d prior to expected parturition; 28 d after the far-off visit), and fresh (from 0 to 21 d in milk (DIM); 16 to 21 d after the close-up visit) periods. The formulated diets fed to the cows observed at the time of the visit were collected from the nutritionist or herd manager. The forages fed to the observed group of cows were sampled at each visit and analyzed by near-infrared spectroscopy at a commercial laboratory (Green Mountain Feed Testing Laboratory, Newport, VT, USA). The formulated diets with analyzed forage samples were input into the Cornell Net Carbohydrate and Protein System (CNCPS, v. 6.1, Cornell University, Ithaca, NY, USA). The diet CNCPS files were imported into the Nutritional Dynamic System Professional (NDS Professional version 3.8.10.06, RUM&N Sas, Reggio Emilia, Italy) for nutrient extraction.

For each visit, the herds were retrospectively dichotomized within parity group (primiparous vs. multiparous cows) into different nutritional strategies, as determined by starch, forage NDF, or both, based on the CNCPS-formulated diet. The CNCPS-formulated diet was used instead of the analyzed TMR as we only collected TMR samples once relative to the visit period and were not always able to collect the TMR sample at the time of feed delivery. For the far-off period, herds were characterized as being fed a controlled energy diet (CE; <16.5% starch and ≥40% forage NDF) or not CE (NCE; ≥16.5% starch or <40% forage NDF or both). For the close-up period, herds were characterized as being fed a higher-forage NDF (HF; ≥40% forage NDF) or lower-forage NDF diet (LF; <40% forage NDF) and for the fresh period, herds were characterized as being fed a lower-starch (LS; <25.5% starch) or higher-starch diet (HS; ≥25.5% starch).

A more complete description of the blood sampling scheme and analysis was provided previously [14]. In brief, a convenience sample of 11 to 24 cows per herd was blood-sampled twice: once during the close-up dry-period visit and once during the fresh-period visit. Approximately one-third of the sampled cows were primiparous cows to reflect herd demographics. Primiparous cows were defined as cows entering their first lactation and multiparous cows were defined as cows entering their second or greater lactation. Postpartum whole blood was analyzed for BHB concentrations. Prepartum and postpartum plasma were analyzed for NEFA concentrations and postpartum plasma was analyzed for Hp concentrations for cows that were 0 to 12 DIM.

The outcomes of interest were: (1) disorder incidence (DI; one or more of displaced abomasum, clinical ketosis, or metritis within 30 DIM), (2) prevalence of elevated prepartum NEFA (≥0.17 mmol/L) for multiparous cows, (3) prevalence of elevated postpartum NEFA (≥0.59 mmol/L), (4) prevalence of postpartum BHB (≥1.2 mmol/L), (5) prevalence of elevated postpartum Hp (≥0.45 g/L), (6) average milk yield at 4 wk of lactation (WK4MP), (7) average 305-d mature equivalent milk yield at the 4th test day (ME305; mean ± standard deviation: 114 ± 13 DIM), (8) 21-d herd pregnancy rate (PR), (9) herd probability of pregnancy (PP), and (10) the pregnancy risk to first service (PRFS). Health disorders were recorded by herd personnel and disorder definitions were defined previously [14]. Biomarker thresholds were chosen as they were the herd-alarm levels associated with an increase in disorder incidence for primiparous and multiparous cows [15]. The prevalence of elevated prepartum NEFA concentrations was only evaluated for multiparous cows since a herd-alarm level was not identified for primiparous cows [15]. Milk yield at 4 wk of lactation and ME305 were acquired from the farm’s Dairy Comp 305 records or DHIA records, provided as a Dairy Comp 305 file. The herd PP was determined by averaging the PP for the first 2 estrus cycles after the herd voluntary waiting period (VWP) for the group of cows that calved within the same calving date range as the cows observed using Dairy Comp 305 software calculations (the percent of services with confirmed pregnancy; [16]). The 21-d PR was determined by averaging the two-21 d periods after the herd VWP for the group of cows that calved within the same time frame as the cows observed using Dairy Comp 305 software calculations (calculated as the heat detection rate * PP; [16]). Cows that were never bred were removed from the PRFS analysis (*n* = 155). All outcomes were calculated by parity within a herd due to multiparous and primiparous cows being fed different diets in some herds.

### 2.2. Statistical Analysis

A sample size calculation was conducted to estimate the prevalence of cows with hyperketonemia within a farm, as described previously [15]; however, this sample size calculation is for cow-level sampling, which is not applicable to group-level outcomes. A sample size calculation was not conducted for assessing differences in outcomes of interest between nutritional strategies due to no previous observational studies assessing transition cow nutritional strategies with our outcomes of interest, nor could we reasonably estimate the variance. Raw data were entered into Microsoft Excel (Microsoft Corp., Redmond, WA, USA). Data-cleaning was conducted to correct human recording errors prior to statistical analysis by double-checking data entry values and observing outliers when performing descriptive statistics.

All statistical analyses were performed in SAS software (SAS 9.4, SAS Institute Inc., Cary, NC, USA). Descriptive statistics were calculated using the FREQ and MEANS procedures. The distribution of herds feeding a commercial anionic supplement during the close-up period, feeding a rumen-protected choline supplement during the close-up and fresh periods, feeding monensin during the close-up and fresh periods, and routinely administering oral propylene glycol before or after parturition to multiparous cows between nutrition strategies within each period was tested with a Fisher’s exact test using PROC FREQ. Factorial ANOVA models were generated using PROC MIXED for all outcomes.

Multiparous and primiparous cows were initially analyzed separately. Nutritional strategies were assessed during the dry period and the periparturient period using two models for each outcome: (A) dry-period model: included the fixed effects of the nutritional strategy during the far-off and close-up dry periods, and (B) periparturient-period model: included the fixed effects of the nutritional strategy during the close-up dry and fresh periods. Calving season (cool (October through April) vs. warm (May through September)) and the interaction between the nutritional strategy fixed effects were offered to the models as covariates but were removed if *p* ≥ 0.20 for the interaction term or if *p* ≥ 0.10 for season using a manual backwards-elimination process.

For all assessed models, if the magnitude of the effect *p*-values and directionality for associations between the nutritional strategies and outcome of interest were similar between parity groups, then data were combined and the full model also included herd as a random effect. Parity group was also offered to the model as a covariate and the full model was reassessed using a manual backwards-elimination process to remove the interaction term if *p* ≥ 0.20, and season and parity group if *p* ≥ 0.10. Parity group was not included in the ME305 model since ME305 is a calculation that accounts for parity group.

If the interaction term had a *p* < 0.20 for the dry- and periparturient-period models, the unadjusted and multiple comparison adjusted *p*-values are reported. For the periparturient models, if an interaction had a *p* < 0.20, then *p*-values were corrected for multiple comparisons using Tukey’s honest significance test, as all comparisons were of interest. For the dry-period models, *p*-values were corrected for multiple comparisons using a Bonferroni test in the LSMESTIMATE statement. Comparisons to NCE far-off- and HF close-up-fed herds were not assessed due to a limited number of observations, as this is not a common nutritional strategy amongst farms in the northeastern United States. For multiple comparisons, unadjusted and adjusted *p*-values are reported to address hypotheses and observational effects that should be investigated more thoroughly in controlled research trials.

Plots of studentized residuals were visually assessed for homogeneity and normality of variance for the mixed effects models and, if observed, extreme outliers were removed. The least squares means (±standard error) are reported throughout for all models. Due to our study being an observational study and the results having on-farm applicability, the nutritional strategy main effects or interactions were identified as being significantly associated with the outcome of interest if *p* < 0.20, which accounts for a 1 in 5 chance that the observed differences were by chance.

## 3. Results

### 3.1. Descriptive Results and Study Population

A more complete description of the study population was reported previously [14,15]. A total of 1473 cows were represented across the 72 farms. There was no evidence of a difference (*p* > 0.10; Table S1) in the distribution of farms feeding a commercial anionic supplement during the close-up dry period, feeding monensin or rumen-protected choline during the close-up dry or fresh periods, or routinely administering oral propylene glycol before or after parturition between nutrition strategies within each period for multiparous cows. The formulated nutrient composition utilizing the analyzed forage composition is reported in Table 1. Means for minerals are not provided since the forages were analyzed by near-infrared spectroscopy. For the far-off dry period, the diets classified as CE had a greater proportion of forage NDF and a lower proportion of starch, net energy for lactation (NE_L_), and metabolizable protein, compared to the diets classified as NCE. For the close-up period, the diets classified as HF had a greater proportion of forage NDF and a lower proportion of starch and ether extract compared to the diets classified as LF. For the fresh period, the nutrient composition was very similar between diets classified as LS and HS, except the proportion of starch and fermentable starch was lower in the diets classified as LS compared to HS.

Primiparous cows were not present in 2 of the 72 herds. Data from primiparous cows in one herd were not analyzed for the prevalence of elevated Hp concentrations because samples were collected >12 DIM. Nineteen herds (15 for the herd prevalence of elevated Hp concentration analysis) were removed from the dry-period nutritional strategy primiparous cow analyses and one herd (except for the herd prevalence of elevated Hp concentration analysis) was removed from the periparturient nutritional strategy primiparous cow analyses due to missing diet information. Six herds were removed from the DI model due to the herds not recording one of the disorders. For the herd prevalence of elevated Hp concentration analysis in primiparous cows, herds were removed if there were <3 primiparous cows observed within a herd (*n* = 12). Two herds were removed from the ME305 analysis and one herd was removed from the milk yield at 4 wk of lactation due to the farm not participating in monthly DHIA test or missing records. For the 21 d PR and PP, four herds were removed due to the farm either using natural service (*n* = 2), compliance in the farm’s reproductive program being compromised (*n* = 1), or not having adequate records (*n* = 1). Three herds were removed from the PRFS analysis due to the farm using natural service or compliance with the farm’s reproductive program being compromised.

### 3.2. Prevalence of Elevated Biomarkers

#### 3.2.1. Prepartum Nonesterified Fatty Acids

The prevalence of elevated prepartum NEFA concentrations outcome was only evaluated for the dry-period nutritional strategy since the outcome was evaluated prior to the fresh period. The associations between nutritional strategies during the dry period and the prevalence of elevated prepartum NEFA concentrations for multiparous cows are reported in Table 2. There was an interaction between far-off and close-up nutritional strategies (*p* = 0.15); however, there was no evidence that the common nutritional strategies differed (unadjusted *p* ≥ 0.39). Multiparous cows in HF-fed herds had a higher prevalence of elevated prepartum NEFA concentrations than LF-fed herds (*p* = 0.14).

#### 3.2.2. Postpartum Nonesterified Fatty Acids

The associations between nutritional strategies and the prevalence of elevated postpartum NEFA concentration are reported in Table 3. Multiparous and primiparous cows were separated for the dry- and periparturient-period nutritional strategy analyses due to dissimilar results. We found no evidence that there was a difference in the prevalence of elevated postpartum NEFA concentrations for the dry-period or periparturient-period nutritional strategies for multiparous cows. There was an interaction between the far-off and close-up nutritional strategies (*p* = 0.17) for primiparous cows; however, there was no evidence that the common nutritional strategies differed (unadjusted *p* ≥ 0.50). Higher-forage NDF-fed herds were associated with a higher prevalence of elevated postpartum NEFA concentrations than LF-fed herds for primiparous cows (*p* = 0.13). There was an interaction between the close-up and fresh-period nutritional strategies for primiparous cows such that herds fed HF × HS had a higher prevalence of elevated NEFA than herds fed LF × HS (unadjusted *p* = 0.03) and HF × LS (unadjusted *p* = 0.18), and herds fed LF × LS had a higher prevalence of elevated postpartum NEFA than LF × HS (unadjusted *p* = 0.14).

#### 3.2.3. β-Hydroxybutyrate

Results for the prevalence of elevated BHB concentration analysis are reported in Table 4. Multiparous and primiparous cows were separated for the dry-period nutritional strategy analysis due to dissimilar results. For the dry-period model for multiparous cows, HF-fed herds were associated with a lower prevalence of elevated BHB concentrations during the close-up period compared to LF-fed herds (*p* = 0.07) and there was no evidence of a difference in the prevalence of elevated BHB concentrations for the far-off nutritional strategies (*p* = 0.59). There was an interaction between the far-off and close-up nutritional strategy for primiparous cows such that CE × LF-fed herds had a higher prevalence of elevated BHB concentrations than NCE × LF-fed herds (unadjusted *p* = 0.12). Primiparous and multiparous cows were combined for the periparturient model due to similar results. Higher-forage NDF-fed herds were associated with a lower prevalence of elevated BHB concentrations than LF-fed herds (*p* = 0.11). Higher-starch-fed herds had a lower prevalence of elevated BHB concentrations than LS-fed herds (*p* = 0.02).

#### 3.2.4. Haptoglobin

Results for the prevalence of elevated Hp concentration analysis are reported in Table 5. For the dry-period nutritional strategy, we found no evidence that there was a difference in the prevalence of elevated Hp concentrations for the far-off nutritional strategy (*p* = 0.77); however, primiparous and multiparous cows in HF-fed herds during the close-up period were associated with a higher prevalence of elevated Hp concentrations than LF-fed herds (*p* = 0.14). We found no evidence that there was a difference in the prevalence of elevated postpartum Hp concentrations for the periparturient-period nutritional strategies for multiparous cows. Primiparous cows in LS-fed herds had a lower prevalence of elevated Hp concentrations that HS-fed herds (*p* = 0.06).

### 3.3. Postpartum Performance Outcomes

#### 3.3.1. Disorder Incidence

The associations between nutritional strategies and DI are reported in Table 6. We found no evidence that there was a difference in DI for the dry-period nutritional strategies for multiparous and primiparous cows. There was an interaction between the close-up and fresh nutritional strategies for multiparous and primiparous cows (*p* = 0.009) such that HF × LS-fed herds had higher DI than HF × HS- (unadjusted *p* = 0.05) and LF × LS (unadjusted *p* = 0.08)-fed herds, and LF × HS-fed herds had higher DI than HF × HS- (unadjusted *p* = 0.05) and LF × LS (unadjusted *p* = 0.08)-fed herds.

#### 3.3.2. Milk Yield

Results for the WK4MP and ME305 analyses are reported in Table 7 and Table 8, respectively. We found no evidence that there was an association between different nutritional strategies and either milk yield outcome.

#### 3.3.3. 21-d Pregnancy Rate

Results from the 21-d PR analysis are reported in Table 9. Multiparous and primiparous cows were separated for the dry-period nutritional strategy analysis due to dissimilar results. For multiparous cows, there was no evidence that the 21-d PR differed between far-off nutritional strategies (*p* = 0.69); however, LF-fed herds were associated with higher 21 d PR during the close-up period compared to HF-fed herds (*p* = 0.14). There was an interaction between the far-off- and close-up-period nutritional strategies for primiparous cows such that CE × HF- (unadjusted *p* = 0.09) and NCE × LF- (unadjusted *p* = 0.12) fed herds had higher 21-d PR than CE × LF-fed herds. Multiparous and primiparous cows were also separated for the periparturient-period nutritional strategy analysis due to dissimilar results. Similar to the dry-period nutritional strategy model for multiparous cows, LF-fed herds were associated with higher 21-d PR than HF-fed herds during the close-up period (*p* = 0.14); however, there was no evidence that the 21-d PR differed between the fresh-period nutritional strategies (*p* = 0.75). We found no evidence that the 21-d PR differed between the close-up- (*p* = 0.49) or fresh-period (*p* = 0.22) nutritional strategies for primiparous cows. Calving season remained in the dry- and periparturient-period models for multiparous cows such that cows that calved during the warmer months had lower 21-d PR than cows that calved during the cool months.

#### 3.3.4. Herd Probability of Pregnancy

The results from the PP analysis are reported in Table 10. Multiparous and primiparous cows were separated for the dry-period nutritional strategy analysis because the results were dissimilar; however, we found no evidence that there was an association between different dry-period nutritional strategies and PP for multiparous cows. For primiparous cows, there was an association for close-up nutritional strategy in the dry-period model such that HF-fed herds were associated with higher PP than LF-fed herds (*p* = 0.17). For multiparous cows, there was no evidence that the PP differed between the periparturient nutritional strategies. For primiparous cows, there was an interaction (*p* = 0.14) between the close-up and fresh-period nutritional strategies such that HF × HS-fed herds had higher PP than HF × LS-fed herds (unadjusted *p* = 0.02), LF × LS-fed herds (unadjusted *p* = 0.007), and LF × HS-fed herds (unadjusted *p* = 0.02).

#### 3.3.5. Pregnancy Risk to First Service

The results for the PRFS analysis are reported in Table 11. Multiparous and primiparous cows were combined in the dry-period and periparturient-period models due to similar results. We found no evidence that there was an association between different nutritional strategies and PRFS.

## 4. Discussion

Our study identified dry-period and periparturient-period nutritional strategy relationships with metabolic- (NEFA and BHB) and inflammation-related (Hp) analytes, DI, milk yield, and reproductive performance. Evaluating nutritional strategies provides dairy nutritionists with guidelines for the best strategy to implement on the farm for cow performance, while allowing for the flexibility to adjust the nutrients to suit the needs of the cows.

To our knowledge, there have not been any large, observational studies evaluating nutritional strategies on transition-period health and performance outcomes on dairy farms; therefore, our discussion will focus on results reported in controlled research trials evaluating the plane of energy or overall nutrient supply. It is important to note major differences between our study and previous controlled research trials. First, one of the biggest differences we observed between the present study and previous controlled research trials was the vast difference in the energy concentration of the treatment strategies in many of the controlled trials. There are moderate differences in the mean nutrient concentration in our defined nutritional strategies (Table 1). Based on the authors’ extensive field involvement and experience with commercial dairies in the northeastern United States, the nutrient concentrations are within the typical use range. It is of note that many controlled studies have tested the limits or have utilized treatments that have discernable differences (e.g., 14% vs. 26% starch) as a way of investigating biological functions; therefore, some of these diets may not be representative of diets typically fed on a farm [17,18]. Secondly, some of these controlled research trials have tested treatments that involved feed restriction, which is not advised on farms as it can alter feeding behavior [19]. For the purpose of this discussion, results from restricted-fed treatments within studies will not be included. Thirdly, our analysis was conducted at the herd-level, whereas, in controlled research trials, it was analyzed at the cow-level; therefore, our outcomes may differ slightly compared to those in the literature. In addition, we are not able to infer possible cow-level biological mechanisms due to the analysis being conducted at the herd-level and missing critical cow-level data, such as individual dry matter intake (DMI); however, herd-level associations between the proportion of cows with elevated analytes and postpartum performance outcomes have been evaluated and discussed previously for this dataset [15]. Lastly, the controlled research trials discussed herein evaluated cows in tiestalls and not freestalls; therefore, the cows observed in this study were subjected to additional environmental factors, such as negative social interactions and increased competition, which may alter the cows’ feeding and lying behavior. This may provide one possible explanation as to why differences were observed in this study compared to controlled research trials. For these reasons, this study provides external validity for controlled research trials.

### 4.1. Dry-Period Nutritional Strategies

In our study, the dry-period nutritional strategy was only associated with the prevalence of elevated prepartum NEFA, postpartum NEFA, BHB and Hp concentrations, 21-d PR and PP outcomes. Since most controlled research trials evaluating nutritional strategies have not evaluated reproduction, reproductive outcomes will be reviewed later in the discussion. Overall, results from our dry-period nutritional strategy models were similar between multiparous and primiparous cows.

Although an interaction between far-off and close-up nutritional strategies was observed for the prevalence of elevated prepartum NEFA concentrations, significant differences between common nutritional strategies were not observed; however, multiparous cows in HF-fed herds had a higher prevalence of elevated prepartum NEFA concentrations compared to LF-fed herds. Similarly, Janovick et al. [4] and Mann et al. [5] reported higher prepartum NEFA concentrations in multiparous cows fed a controlled-energy dry-period diet compared to a high-energy dry-period diet. In another study observing primiparous and multiparous cows fed a controlled-energy far-off diet and either a controlled-energy or high-energy close-up diet, Vasquez, et al. [20] also reported high prepartum NEFA concentrations in cows fed the controlled-energy close-up diet.

Contrary to our hypothesis, an interaction between far-off and close-up nutritional strategies was not observed for the prevalence of elevated postpartum NEFA concentrations for multiparous cows. In addition, an interaction between far-off and close-up nutritional strategies on the prevalence of elevated postpartum NEFA concentrations was observed for primiparous cows, although significant differences between common nutritional strategies were not observed; however, HF-fed herds had a higher prevalence of elevated postpartum NEFA concentrations than LF-fed herds. Contrary to our results, previous studies reported lower postpartum NEFA concentrations for cows fed a controlled-energy diet compared to a high-energy diet [4,5,6,20]. Our analysis was at the herd-level and not the cow-level; therefore, it is plausible that if the unit of observation was the cow, we may have detected differences in postpartum NEFA concentrations for multiparous cows. Our herd-level analysis evaluated if there was a difference in the proportion of cows above the identified threshold.

For multiparous cows, an interaction between far-off and close-up nutritional strategies on the prevalence of elevated BHB concentrations was not observed; however, there was a greater prevalence of elevated BHB concentrations in herds where multiparous cows were fed an LF close-up diet compared to a HF diet. For primiparous cows, there was an interaction between the far-off and close-up strategy such that CE × HF- and NCE × LF-fed herds had a similar prevalence of elevated BHB concentrations; however, only NCE × LF-fed herds had a statistically lower prevalence of elevated BHB concentrations than CE × LF-fed herds. These results support controlled research findings for multiparous cows showing that feeding a controlled-energy close-up diet can minimize increases in postpartum BHB concentrations [4,5,6]; however, results for primiparous cows are not consistent. Richards et al. [6] observed greater postpartum BHB concentrations in primiparous and multiparous cows fed a high-energy diet throughout the dry period compared to cows fed a controlled-energy diet throughout the dry period or a controlled-energy far-off followed by a high-energy close-up diet. Similarly, Janovick et al. [4] observed greater postpartum BHB concentrations in primiparous cows fed a high-energy dry-period diet compared to a controlled-energy dry-period diet.

Contrary to our hypothesis, an interaction between far-off and close-up nutritional strategies was not observed for the prevalence of elevated Hp concentrations for primiparous or multiparous cows, although cows in HF-fed herds had a higher prevalence of elevated Hp concentrations than LF-fed herds. To our knowledge, Hp concentrations have not been evaluated in controlled research trials evaluating prepartum nutritional strategies and should be evaluated in the future to explore possible biological mechanisms.

An association was not observed between far-off and close-up nutritional strategies and DI. Although caution should be used when interpreting health data in controlled research trials due to sample size, Mann et al. [5] reported half as many hyperketonemia cases and no clinical ketosis cases within 3 wk postpartum for cows fed a controlled-energy diet during the entire prepartum period compared to cows fed an intermediate (controlled-energy far-off diet and intermediate-energy close-up diet) or high-energy diet (high energy during the entire prepartum period). Janovick et al. [4] observed an increased incidence of displaced abomasum and ketosis, and had more cows with more than 1 negative health event, regardless of parity, for cows fed the high-energy prepartum diet for the entire dry period compared to cows fed the controlled-energy diet for the entire dry period. On the contrary, there were not any discernable differences in negative health events between cows that were fed a controlled-energy diet for the whole dry period, a step-up diet (controlled-energy at dry-off, switching to high-energy 21 d before expected parturition), or a high-energy diet for the whole dry period in the study by Richards et al. [6].

As hypothesized, there was not an association between far-off and close-up nutritional strategies on milk yield (WK4MP or ME305). Our results are similar to previous studies [4,5,6,8], which did not report a difference in milk yield between different dry-period nutritional strategies.

In general, previous studies investigating transition cow nutritional strategies have not considered the fresh diet when investigating dry-period nutritional effects on postpartum performance, metabolic, and health outcomes. Most previous studies had cows transition onto a higher-energy fresh cow diet [4,6,8,20]; however, Mann et al. [5] had cows transitioning onto a lower-energy fresh cow diet (21.2% starch, 35.4% NDF). In addition, these previous studies only considered the overall dry-period strategy (i.e., the interaction between the far-off and close-up strategy) as the treatment and did not investigate the far-off and close-up nutritional strategies in a 2 × 2 factorial study design, which would have been a more similar comparison to the current observational study design.

### 4.2. Periparturient-Period Nutritional Strategies

The periparturient-period nutritional strategy models identified close-up and fresh-period nutritional strategy associations with the prevalence of elevated postpartum NEFA and Hp concentrations for primiparous cows, prevalence of elevated postpartum BHB concentrations, DI, PP for primiparous cows, and PR for multiparous cows. This section of the discussion will focus on the few controlled research studies that have evaluated the effects of the interaction between close-up and fresh-period nutritional strategies. As stated previously, most controlled research trials evaluating nutritional strategies have not evaluated reproduction; therefore, we will review reproductive outcomes later in the discussion.

In the current study, an interaction between the close-up and fresh nutritional strategies was observed for the prevalence of elevated postpartum NEFA concentrations such that primiparous cows in herds fed a HF × HS diet had a higher prevalence of elevated NEFA concentrations than LF × HS- or HF × LS-fed herds and LF × LS-fed herds had a higher prevalence than LF × HS-fed herds. In a 2 × 2 factorial design, Rabelo et al. [17] fed multiparous and primiparous cows a low-energy (1.58 Mcal/kg NE_L_, 39.7% NDF, and 38.2% non-fiber carbohydrate (NFC)) or high-energy (1.70 Mcal/kg NE_L_, 32.2% NDF, 44.6% NFC) close-up dry-period diet and a low-energy (1.57 Mcal/kg NE_L_, 29.9% NDF, and 41.1% NFC) or high-energy (1.63 Mcal/kg NE_L_, 24.9% NDF, 47.2% NFC) fresh-period diet. The authors observed a main effect of close-up nutritional strategy on postpartum NEFA concentrations: cows fed the high-energy prepartum diet had lower NEFA concentrations than cows fed a low-energy prepartum diet. The authors did not observe an interaction between the close-up and fresh strategy for postpartum NEFA concentrations, unlike the present study; however, both prepartum treatments in the Rabelo et al. [12,17] study were much higher in NE_L_ and NFC concentrations than in our study. In another 2 × 2 factorial design, Haisan et al. [18] fed multiparous and primiparous cows a control (14.0% starch, 47.7% NDF) or high-starch (26.1% starch, 37.8% NDF) close-up diet and a high-fiber (25.1% starch, 33.8% NDF) or high-starch (32.8% starch, 27.2% NDF) fresh diet. The authors did not observe an interaction between close-up and fresh-period nutritional strategies; however, there was a main effect of close-up and fresh-period nutritional strategy such that cows fed the high-starch prepartum or postpartum diet had higher postpartum NEFA concentrations than the cows on the control prepartum or high-fiber postpartum treatment.

In our periparturient model, an association between the main effect of close-up strategy and the prevalence of elevated BHB concentrations was observed, such that cows in LF-fed herds had a higher prevalence than HF-fed herds. Similar to our results, Haisan et al. [18] observed an association between the close-up strategy and BHB concentrations at 10 DIM such that cows fed the high-starch close-up diet had greater BHB concentrations than cows fed the control close-up diet; however, Rabelo et al. [17] did not observe an effect of close-up nutritional strategy on postpartum BHB concentrations.

We also observed an interaction between the close-up and fresh nutritional strategies for DI such that herds that fed cows a HF × HS or LF × LS diet had a lower DI compared to herds that fed cows a HF × LS or LF × HS diet. Contrary to our findings, Haisan et al. [18] reported a greater incidence of negative health events in cows fed the high-starch prepartum and postpartum diets, but there was not a discernable difference amongst the other nutritional strategy combinations. It is important to note that there are vast differences in the starch concentration of the high-starch close-up and fresh diets reported by Haisan et al. [18] compared to the current study; therefore, the high-starch close-up and high-starch fresh strategy does not adequately reflect any of the strategies observed in our study. As stated before, caution needs to be used when interpreting health event data from controlled research trials due to a limited sample size. The study by Haisan et al. [18] is the only study that reported health event data when investigating periparturient nutritional strategy effects.

Contrary to our hypothesis, an association was not observed between the interaction of close-up and fresh nutritional strategies and milk yield outcomes (WK4MP or ME305). Rabelo et al. [12] did not observe any treatment effects on milk yield through 20 DIM; however, Haisan et al. [18] reported greater milk yield through 20 DIM in cows fed the control prepartum diet and high-starch postpartum compared to any other treatment. We also hypothesized that cows fed HF close-up and HS fresh diets would have an increased prevalence of elevated Hp concentrations compared to cows fed HF close-up and LS fresh diets; however, an association between periparturient nutritional strategies and the prevalence of elevated Hp concentrations was not observed. Haisan et al. [18] did not observe an interaction between prepartum and postpartum nutritional strategies on Hp concentrations; however, cows fed the high-starch postpartum diet had lower Hp concentrations than cows fed the high-fiber postpartum diet.

Our results and others [12,18] indicate that the interaction between the close-up and fresh diet should be considered when evaluating certain outcomes.

### 4.3. Fresh-Period Nutritional Strategies

In the periparturient models, the main effect of fresh-period nutritional strategy was associated with the prevalence of elevated BHB concentrations for all cows and the prevalence of elevated Hp concentrations for primiparous cows. In agreement with our hypothesis, herds with cows fed a higher-starch fresh diet had a lower prevalence of elevated BHB concentrations than herds with cows fed a lower-starch fresh diet. McCarthy et al. [13,21] evaluated dietary starch in the fresh period and fed a high-starch (26.2% starch, 34.3% NDF, 1.64 Mcal/kg NE_L_) or low-starch (21.5% starch, 36.9% starch, 1.56 Mcal/kg NE_L_) diet through 21 DIM before all cows were fed the high-starch diet through 63 DIM. Supporting our results, the authors observed lower BHB concentrations through 21 DIM, regardless of parity, in cows fed the high-starch diet compared to the low-starch diet. Rabelo et al. [17] also observed a postpartum treatment by time interaction such that BHB concentrations were lower for cows fed the high-starch postpartum diet at 7 and 21 DIM. Our results and others [17,21] support the notion that increasing dietary starch immediately after parturition, rather than delaying, as in a step-up fresh approach, is effective in minimizing the increase in BHB concentrations; however, these results are not consistent in the literature. Dann and Nelson [22] did not observe a difference in BHB concentrations for the first 3 wk of lactation when feeding a low-starch (21.0% starch through 91 DIM), medium-to-high-starch (23.2% starch for 21 DIM then 25.5% until 91 DIM) or high-starch (25.5% starch through 91 DIM) postpartum diet. Sun and Oba [23] also did not observe treatment or a treatment by week interaction for BHB concentrations when feeding high-starch (29.2% starch) or low-starch (19.1%) diets from parturition through 12 wk of lactation.

In agreement with our hypothesis, herds that fed primiparous cows an HS fresh diet had a higher prevalence of elevated Hp concentrations than herds that fed primiparous cows an LS fresh diet. Supporting our results, McCarthy et al. [21] observed higher Hp concentrations through 15 DIM in cows fed a high-starch compared to a low-starch diet, regardless of parity; however, Haisan et al. [18] observed the opposite results. Regardless of parity, Haisan et al. [18] reported lower serum Hp concentrations in cows fed the high-starch fresh diet compared to the high-fiber fresh diet; however, it is important to note that the high-fiber diet was very similar to the high-starch diet in the study by McCarthy et al. [21].

Contrary to our hypothesis, we did not observe an association between fresh-period nutritional strategy and the prevalence of elevated postpartum NEFA concentrations, milk yield (WK4MK or ME305), or DI. Dann and Nelson [22] reported higher NEFA concentrations in cows fed the medium-starch diet through 21 DIM compared to the high-starch diet, yet greater milk yield in cows fed the low-starch diet compared to the high-starch diet. McCarthy et al. [21] reported lower NEFA concentrations, no difference in the frequency of health events, and greater early-lactation milk yield [13] for cows fed a high-starch fresh diet compared to a low-starch diet. Sun and Oba [23] reported higher NEFA concentrations for primiparous (wk 2, 6, and 8 postpartum) and multiparous cows (wk 3 and 4) fed the low-starch postpartum diet compared to the high-starch diet. It is important to note that cows fed the low-starch postpartum diet stayed on that diet through 12 wk of lactation instead of transitioning onto a higher-energy diet earlier in lactation to maintain milk yield, as typically observed in a step-up fresh approach. Interestingly, Sun and Oba [23] did not observe a difference in milk yield between treatments. Although we did not observe any associations with these outcomes, discrepancies amongst the literature when evaluating the starch concentration during the fresh period may be due to differences in the prepartum or postpartum diets. It has been proposed that hypophagic effects may be observed when providing highly fermentable starch sources in fresh diets [10,24]. However, research within the last decade would indicate there might be an interaction between starch or starch digestibility with forage or forage NDF levels on postpartum performance and health [25]. Favorable responses have been observed when feeding a higher-starch or starch digestibility fresh diet with higher forage or forage NDF concentrations [13,21,26] while neutral or negative responses have been observed when feeding a higher-starch fresh diet with lower forage or forage NDF concentrations [18,22,23,24]. Results from a study by Tebbe and Weiss [27] suggest that primiparous cows may benefit from a fresh diet with higher starch and lower forage NDF; however, this study lacked a higher-starch and forage NDF fresh diet treatment, prompting the need for further investigation.

### 4.4. Nutritional Strategies on Reproductive Outcomes

Very limited research has evaluated the effects of nutritional strategies pertaining to energy on reproductive outcomes. This may be due to the limited sample size within the study or the need for an increase in follow-up time; however, Cardoso et al. [28] pooled results within their group from seven studies evaluating prepartum nutrition to evaluate the effects of nutrition on reproductive performance (*n* = 354 multiparous cows, 54 primiparous cows). Far-off- and close-up-period nutritional strategies were assigned as controlled-energy (≤100% of NE_L_ requirements) or high-energy (>100% of NE_L_ requirements). All the lactating diets supplied to the cows in the Cardoso et al. [28] study appeared to be similar and were higher-energy diets (≥1.67 Mcal/kg NE_L_). Cardoso et al. [28] reported less days to pregnancy when cows were fed a controlled-energy diet during the close-up period compared to the high-energy close-up diet; however, there were no differences in days to pregnancy when evaluating the effects of far-off-period nutrition. Similar to Cardoso et al. [28], Vickers et al. [7] reported greater odds of pregnancy at 120 and 150 DIM for cows fed a controlled-energy far-off and close-up diet (13.6% starch, 48.4% NDF, 1.41 Mcal/kg NE_L_) compared to cows fed the controlled-energy far-off and a higher energy close-up diet (16.3% starch, 41.3% NDF, 1.45 Mcal/kg NE_L_). These results correspond to our data for primiparous cows, but not for multiparous cows. In our study, herds fed a CE × HF or NCE × LF dry-period strategy had a higher 21-d PR for primiparous cows than CE × LF-fed herds. For multiparous cows, an interaction was not observed between far-off and close-up nutritional strategies; however, LF close-up-fed herds had a higher 21-d PR than HF-fed herds. In addition, an interaction between close-up and fresh nutritional strategies was observed for primiparous cows such that herds that fed a HF × HS diet had a higher PP than all other nutritional strategy combinations. It is important to note that we observed an interaction between far-off and close-up nutritional strategy for primiparous cows, while Cardoso et al. [28] did not evaluate the interaction between far-off and close-up nutrition.

It has been suggested that feeding controlled-energy diets during the prepartum period will prevent the over-consumption of nutrients during the prepartum period and promote DMI during the early postpartum period, thus reducing the degree of negative energy balance compared to feeding high-energy prepartum diets [9,29]. The severity of negative energy balance in early lactation has been associated with pre- and postovulatory reproductive failure as it coincides with follicular development and uterine involution [30]. Follicles and oocytes will ovulate from 50 to 60 d after development in the early lactation period [29]; therefore, nutrition during the early postpartum period can play a critical role in determining if ovulation occurs after the herd voluntary waiting period. Although research on optimizing reproductive performance through postpartum nutritional strategies is limited, glucose is required for oocyte maturation and the development of the blastocyst and it has been suggested to feed more starch (more glucogenic diet) to increase blood insulin and reestablish the growth hormone-insulin-like growth factor 1 axis to resume ovarian activity [9,29].

### 4.5. Limitations and Strengths

Although we intended to adequately classify these herds into different nutritional strategies, there are many limitations to this study. In this study, we focused on starch and forage inclusion as a proxy for energy intake but we did not account for other nutrients, such as amino acids, that may provide substrates for the tricarboxylic acid cycle, or vitamins and minerals that may have an impact on immune function and reproductive performance. Our results may have been confounded if the dietary cation–anion difference varied greatly across nutritional strategies; however, we do not believe this to be an issue as commercial anionic supplements were included in the majority of close-up diets for multiparous cows (77.8% of farms [14]) and there was not a difference in the distribution of herds implementing commercial anionic supplements between the nutritional strategies within each period. Individual or pen-level DMI, which would have provided additional information with regard to energy balance, could also be a confounder that was not accounted for due to herd-recording limitations. Forage quality was not accounted for, which may influence DMI, such as the forage chop length and TMR particle size distribution, moisture, or if the forage was spoiled or moldy. The time spent on each of these diets was also not accounted for. Particularly in the fresh period, cows may have been on the fresh cow diet for 14 to 30 d, which may limit intake depending on the concentration of forage NDF [31]. We also did not assess the overall nutritional strategy of the herd as we were not powered for that many comparisons. Despite these limitations, this observational study was performed using a large number of freestall, commercial dairy herds versus tiestall herds, allowing for the influence of other environmental factors. We were able to evaluate if current nutritional strategy recommendations are supported in the field, despite the variation observed with other management factors.

Nutritional strategies are multifactorial and are likely driven by a wide range of factors, such as the body condition score of the cow, the interaction between nutrients in the diet, and social and environmental factors. These factors may explain why one strategy may work well on one farm but lead to an increase in fresh cow health disorders or decreased milk yield in other herds. As stated by Van Saun and Sniffen [32], “In the current transition cow system, a range of feeding program and grouping strategies are observed, with no one approach consistently resulting in the desired outcome”.

## 5. Conclusions

These results can be applied to farms feeding typical forages and diets observed in the northeastern United States. In general, the results of our study support feeding multiparous cows a higher-forage NDF close-up and higher-starch fresh diet to minimize the excessive prevalence of elevated BHB concentrations and reduce disorder incidence in the early postpartum period. Similar to multiparous cows, the results of our study support feeding primiparous cows a controlled energy far-off, higher-forage NDF close-up, and higher-starch fresh diet to maximize reproductive performance, minimize the excessive prevalence of elevated BHB concentrations, and reduce disorder incidence in the early postpartum period.

## Figures and Tables

**Table 1:** Formulated nutrient composition (mean ± SD, % of dry matter (DM), unless otherwise noted) for the nutritional strategies during the far-off dry, close-up dry, and fresh periods for diets fed to primiparous cows, multiparous cows, or both. The formulated diets with analyzed forage samples were inputted into the Cornell Net Carbohydrate and Protein System (CNCPS v. 6.1, Cornell University, Ithaca, NY, USA) and CNCPS files were imported into Nutritional Dynamic System Professional (NDS Professional version 3.8.10.06, RUM&N Sas, Reggio Emilia, Italy) for nutrient extraction.

	Far-Off ^1^		Close-Up ^2^		Fresh ^3^	
Nutrient ^4^	CE	NCE	HF	LF	LS	HS
DM, % of as fed	37.8 ± 5.3	45.3 ± 6.1	42.9 ± 6.3	45.9 ± 5.7	44.9 ± 4.2	45.8 ± 4.2
CP	13.4 ± 2.0	14.0 ± 1.4	13.8 ± 1.1	14.8 ± 1.5	16.5 ± 0.9	16.3 ± 0.9
Soluble protein, % CP	49.4 ± 8.0	38.3 ± 6.9	39.1 ± 6.2	37.0 ± 6.6	36.3 ± 5.0	38.1 ± 5.0
Acid detergent fiber	32.9 ± 2.2	27.3 ± 2.0	29.4 ± 1.5	26.0 ± 2.2	20.6 ± 1.3	19.8 ± 1.3
aNDFom	49.9 ± 3.3	43.3 ± 2.7	46.6 ± 1.9	41.3 ± 3.5	32.9 ± 1.8	31.2 ± 2.1
Forage NDF	48.3 ± 3.8	38.7 ± 3.8	42.7 ± 2.0	34.8 ± 3.4	24.5 ± 1.9	23.6 ± 2.2
Starch	11.8 ± 3.4	17.5 ± 3.9	15.9 ± 2.3	18.5 ± 2.5	23.7 ± 1.4	28.0 ± 1.5
Sugar	2.9 ± 0.8	3.3 ± 1.1	3.3 ± 1.0	3.4 ± 1.0	4.8 ± 1.4	3.7 ± 1.0
NFC	25.2 ± 3.9	30.7 ± 2.7	28.2 ± 2.5	30.6 ± 2.8	37.5 ± 1.6	40.1 ± 1.7
Fermentable starch	7.8 ± 2.6	9.8 ± 2.9	9.4 ± 1.9	10.3 ± 2.0	19.3 ± 3.1	23.4 ± 3.8
Fermentable NDF	13.7 ± 2.5	10.3 ± 2.0	11.2 ± 2.1	9.7 ± 1.7	12.0 ± 1.6	11.0 ± 1.8
Fermentable total carbohydrate	27.1 ± 4.2	25.4 ± 4.5	25.6 ± 4.0	24.8 ± 3.8	39.8 ± 5.4	41.8 ± 5.7
Ether extract	3.28 ± 0.40	3.20 ± 0.52	2.95 ± 0.28	3.61 ± 0.81	5.05 ± 0.71	5.15 ± 0.61
Total fatty acids	1.95 ± 0.25	2.19 ± 0.38	1.99 ± 0.21	2.59 ± 0.64	4.04 ± 0.67	4.17 ± 0.56
NE_L_, Mcal/kg	1.30 ± 0.05	1.37 ± 0.06	1.32 ± 0.06	1.38 ± 0.05	1.59 ± 0.04	1.61 ± 0.04
ME, Mcal/kg of DM	2.02 ± 0.09	2.13 ± 0.09	2.06 ± 0.10	2.15 ± 0.08	2.47 ± 0.06	2.50 ± 0.07
MP, g/kg of DM	70.87 ± 5.62	86.65 ± 7.49	84.43 ± 5.67	91.67 ± 7.94	108.68 ± 6.22	106.58 ± 6.73

^1^ Far-off-period diet characterized as a controlled-energy (CE; <16.5% starch, ≥40% forage NDF; *n* = 29 diets fed to primiparous cows and 43 diets fed to multiparous cows) or not-CE diet (NCE; ≥16.5% starch, <40% forage NDF or both; *n* = 22 diets fed to primiparous cows and 29 diets fed to multiparous cows). ^2^ Close-up-period diet characterized as a higher-forage NDF (HF; ≥40% forage NDF; *n* = 23 diets fed to primiparous cows and 25 diets fed to multiparous cows) or lower-forage NDF diet (LF; <40% forage NDF; *n* = 47 diets fed to primiparous cows and 47 diets fed to multiparous cows). ^3^ Fresh-period diet was characterized as a lower-starch (LS; <25.5% starch; *n* = 30 diets fed to primiparous cows and 32 diets fed to multiparous cows) or higher-starch diet (HS; ≥25.5% starch; *n* = 39 diets fed to primiparous cows and 40 diets fed to multiparous cows). ^4^ CP = crude protein; aNDFom = amylase neutral detergent fiber organic matter; NDF = neutral detergent fiber; NFC = non-fiber carbohydrates NE_L_ = net energy for lactation; ME = metabolizable energy; MP = metabolizable protein.

**Table 2:** Least squares means ± SE of the herd prevalence of elevated prepartum nonesterified fatty acids (NEFA) concentration (proportion of multiparous cows within a herd with prepartum NEFA concentration ≥0.17 mmol/L) for the dry-period nutritional strategy for multiparous cows (*n* = 72 observations) enrolled in a 72-farm prospective cohort study in the northeastern US.

Variable	*n*	Prevalence of Elevated NEFA	*p*-Value
Far-off strategy ^1^			0.66
CE	43	44.1 ± 3.7	
NCE	29	46.7 ± 4.7	
Close-up strategy ^2^			0.14
HF	25	49.8 ± 4.9	
LF	47	41.0 ± 3.4	
Far-off × close-up ^3^			0.15
CE × HF	16	44.2 ± 5.8	
CE × LF	27	43.9 ± 4.5	
NCE × HF	9	55.5 ± 7.8	
NCE × LF	20	38.0 ± 5.2	

^1^ Far-off-period diet characterized as a controlled-energy (CE; <16.5% starch, ≥40% forage neutral detergent fiber (NDF)) or not-CE diet (NCE; ≥16.5% starch, <40% forage NDF or both). ^2^ Close-up period diet characterized as a higher-forage NDF (HF; ≥40% forage NDF) or lower-forage NDF diet (LF; <40% forage NDF). ^3^ Means were compared with a Bonferroni test and comparisons to herds that fed NCE during the far-off period and HF in the close-up period were not assessed due to the limited number of observations, as this is not a common nutritional strategy amongst farms in the northeastern United States.

**Table 3:** Least squares means ± SE of the herd prevalence of elevated postpartum nonesterified fatty acids (NEFA) concentration (proportion of cows within a herd with postpartum NEFA concentration ≥ 0.59 mmol/L) for the dry period (*n* = 72 multiparous observations; *n* = 51 primiparous observations) and periparturient period (*n* = 72 multiparous observations; *n* = 69 primiparous observations) nutritional strategies for multiparous cows and primiparous cows enrolled in a 72-farm prospective cohort study in the northeastern US.

Variable	*n*	Prevalence of Elevated NEFA	*p*-Value
Dry-period model for multiparous cows			
Far-off strategy ^1^			0.92
CE	43	28.9 ± 3.7	
NCE	29	29.5 ± 4.5	
Close-up strategy ^2^			0.62
HF	25	27.7 ± 4.8	
LF	47	30.6 ± 3.5	
Dry-period model for primiparous cows			
Far-off strategy			0.47
CE	29	20.1 ± 4.4	
NCE	22	25.5 ± 6.0	
Close-up strategy			0.13
HF	17	28.5 ± 6.2	
LF	34	17.1 ± 4.0	
Far-off × close-up ^3^			0.17
CE × HF	12	20.7 ± 6.8	
CE × LF	17	19.5 ± 5.7	
NCE × HF	5	36.3 ± 10.5	
NCE × LF	17	14.6 ± 5.7	
Periparturient-period model for multiparous cows			
Close-up strategy			0.64
HF	25	27.6 ± 4.7	
LF	47	30.3 ± 3.4	
Fresh strategy ^4^			0.59
LS	32	27.5 ± 4.2	
HS	40	30.5 ± 3.8	
Periparturient-period model for primiparous cows			
Close-up strategy			0.34
HF	23	22.4 ± 4.7	
LF	46	16.8 ± 3.4	
Fresh strategy			0.83
LS	30	19.0 ± 4.2	
HS	39	20.2 ± 3.9	
Close-up × fresh			0.05
HF × LS	11	16.1 ± 6.7 ^bcXY^	
HF × HS	12	28.7 ± 6.5 ^aX^	
LF × LS	19	21.9 ± 5.1 ^abXY^	
LF × HS	27	11.7 ± 4.3 ^cY^	

^a–c^ Means with different superscripts differ based on unadjusted *p*-value comparisons (*p* < 0.20). ^X–Y^ Means with different superscripts differ based on Tukey’s honest significance test and (*p* < 0.20). ^1^ Far-off-period diet characterized as a controlled-energy (CE; <16.5% starch, ≥40% forage neutral detergent fiber (NDF)) or not-CE diet (NCE; ≥16.5% starch, <40% forage NDF or both). ^2^ Close-up-period diet characterized as a higher-forage NDF (HF; ≥40% forage NDF) or lower-forage NDF diet (LF; <40% forage NDF). ^3^ Means were compared with a Bonferroni test and comparisons to herds that fed NCE during the far-off period and HF in the close-up period were not assessed due to the limited number of observations, as this is not a common nutritional strategy amongst farms in the northeastern United States. ^4^ Fresh-period diet characterized as a lower-starch (LS; <25.5% starch) or higher-starch diet (HS; ≥25.5% starch).

**Table 4:** Least squares means ± SE of the herd prevalence of elevated postpartum BHB concentration (proportion of cows within a herd with BHB concentration ≥ 1.2 mmol/L) for the dry-period (*n* = 72 multiparous observations; *n* = 51 primiparous observations) and periparturient-period (*n* = 141 observations) nutritional strategies for multiparous cows and primiparous cows enrolled in a 72-farm prospective cohort study in the northeastern US.

Variable	*n*	Prevalence of Elevated BHB	*p*-Value
Dry-period model for multiparous cows			
Far-off strategy ^1^			0.59
CE	43	18.2 ± 2.8	
NCE	29	15.9 ± 3.4	
Close-up strategy ^2^			0.07
HF	25	13.0 ± 3.6	
LF	47	21.1 ± 2.6	
Calving season ^3^			0.002
Warm	39	23.9 ± 2.9	
Cool	33	10.2 ± 3.2	
Dry-period model for primiparous cows			
Far-off strategy			0.96
CE	29	11.5 ± 3.3	
NCE	22	11.2 ± 4.5	
Close-up strategy			0.79
HF	17	12.1 ± 4.7	
LF	34	10.6 ± 3.0	
Far-off × close-up ^4^			0.10
CE × HF	12	7.6 ± 5.1 ^ab^	
CE × LF	17	15.4 ± 4.3 ^a^	
NCE × HF	5	16.7 ± 7.9	
NCE × LF	17	5.8 ± 4.3 ^b^	
Periparturient-period model for multiparous and primiparous cows			
Close-up strategy			0.11
HF	48	11.1 ± 2.8	
LF	93	16.6 ± 2.0	
Fresh strategy ^5^			0.02
LS	62	17.8 ± 2.5	
HS	79	10.0 ± 2.3	
Calving season			0.009
Warm	76	18.3 ± 2.3	
Cool	65	9.4 ± 2.2	
Parity			<0.001
Multiparous	72	18.3 ± 2.1	
Primiparous	69	9.4 ± 2.2	

^a–b^ Means with different superscripts differ based on unadjusted *p*-value comparisons (*p* < 0.20). ^1^ Far-off-period diet characterized as a controlled-energy (CE; <16.5% starch, ≥40% forage neutral detergent fiber (NDF)) or not-CE diet (NCE; ≥16.5% starch, <40% forage NDF or both). ^2^ Close-up-period diet characterized as a higher-forage NDF (HF; ≥40% forage NDF) or lower-forage NDF diet (LF; <40% forage NDF). ^3^ Calving season was dichotomized into warm (May through September) vs. cool (October through April). ^4^ Means were compared with a Bonferroni test and comparisons to herds that fed NCE during the far-off period and HF in the close-up period were not assessed due to the limited number of observations, as this is not a common nutritional strategy amongst farms in the northeastern United States. ^5^ Fresh-period diet characterized as a lower-starch (LS; <25.5% starch) or higher-starch diet (HS; ≥25.5% starch).

**Table 5:** Least squares means ± SE of the herd prevalence of elevated postpartum haptoglobin (Hp) concentration (proportion of cows within a herd with Hp concentration ≥ 0.45 g/L) for the dry-period (*n* = 114 observations) and periparturient-period nutritional strategies for multiparous (*n* = 72 observations) and primiparous (*n* = 57 observations) cows enrolled in a 72-farm prospective cohort study in the northeastern US.

Variable	*n*	Prevalence of Elevated Hp	*p*-Value
Dry-period model for multiparous and primiparous cows			
Far-off strategy ^1^			0.77
CE	67	47.7 ± 2.8	
NCE	47	49.0 ± 3.4	
Close-up strategy ^2^			0.14
HF	41	51.6 ± 3.6	
LF	73	45.0 ± 2.7	
Parity			0.002
Multiparous	72	41.4 ± 2.7	
Primiparous	57	55.3 ± 3.4	
Periparturient-period model for multiparous cows			
Close-up strategy			0.36
HF	25	43.1 ± 3.9	
LF	47	38.6 ± 2.9	
Fresh strategy ^3^			0.76
LS	32	40.1 ± 3.5	
HS	40	41.6 ± 3.2	
Periparturient-period model for primiparous cows			
Close-up strategy			0.32
HF	19	57.1 ± 5.7	
LF	38	50.1 ± 4.1	
Fresh strategy			0.06
LS	26	47.2 ± 5.0	
HS	31	59.9 ± 4.6	

^1^ Far-off-period diet characterized as a controlled-energy (CE; <16.5% starch, ≥40% forage neutral detergent fiber (NDF)) or not-CE diet (NCE; ≥16.5% starch, <40% forage NDF or both). ^2^ Close-up-period diet characterized as a higher-forage NDF (HF; ≥40% forage NDF) or lower-forage NDF diet (LF; <40% forage NDF). ^3^ Fresh-period diet characterized as a lower-starch (LS; <25.5% starch) or higher-starch diet (HS; ≥25.5% starch).

**Table 6:** Least squares means ± SE of disorder incidence (incidence of one of more displaced abomasum, clinical ketosis, metritis within 30 d in milk; calculated by parity within herd) for the dry-period (*n* = 111 observations) and periparturient-period (*n* = 129 observations) nutritional strategies for multiparous and primiparous cows enrolled in a 72-farm prospective cohort study in the northeastern US.

Variable	*n*	Disorder Incidence, %	*p*-Value
Dry-period model for multiparous and primiparous cows			
Far-off strategy ^1^			0.99
CE	67	14.0 ± 2.2	
NCE	44	14.0 ± 2.7	
Close-up strategy ^2^			0.67
HF	38	13.2 ± 3.0	
LF	73	14.8 ± 2.1	
Calving season ^3^			0.04
Warm	56	17.7 ± 2.5	
Cool	55	10.3 ± 2.6	
Periparturient-period model for multiparous and primiparous cows			
Close-up strategy			0.94
HF	44	13.2 ± 2.9	
LF	85	13.4 ± 2.1	
Fresh strategy ^4^			0.56
LS	58	14.3 ± 2.6	
HS	71	12.2 ± 2.5	
Close-up × fresh			0.009
HF × LS	23	18.9 ± 4.0 ^aX^	
HF × HS	21	7.4 ± 4.1 ^bY^	
LF × LS	35	9.7 ± 3.2 ^bXY^	
LF × HS	50	17.1 ± 2.7 ^aX^	
Calving season			0.03
Warm	68	17.1 ± 2.4	
Cool	61	9.5 ± 2.5	

^a–b^ Means with different superscripts differ based on unadjusted *p*-value comparisons (*p* < 0.20). ^X–Y^ Means with different superscripts differ based on Tukey’s honest significance test and (*p* < 0.20). ^1^ Far-off-period diet characterized as a controlled-energy (CE; <16.5% starch, ≥40% forage neutral detergent fiber (NDF)) or not-CE diet (NCE; ≥16.5% starch, <40% forage NDF or both). ^2^ Close-up-period diet characterized as a higher-forage NDF (HF; ≥40% forage NDF) or lower-forage NDF diet (LF; <40% forage NDF). ^3^ Calving season was dichotomized into warm (May through September) versus cool (October through April). ^4^ Fresh-period diet characterized as a lower-starch (LS; <25.5% starch) or higher-starch diet (HS; ≥25.5% starch).

**Table 7:** Least squares means ± SE of the herd average milk yield at 4 wk of lactation (WK4MP) for the dry-period (*n* = 122 observations) and periparturient-period (*n* = 139 observations) nutritional strategies for multiparous and primiparous cows enrolled in a 72-farm prospective cohort study in the northeastern US.

Variable	*n*	WK4MP, kg/d	*p*-Value
Dry-period model for multiparous and primiparous cows			
Far-off strategy ^1^			0.76
CE	71	40.0 ± 0.5	
NCE	51	40.2 ± 0.6	
Close-up strategy ^2^			0.28
HF	42	40.5 ± 0.7	
LF	80	39.6 ± 0.5	
Parity			<0.001
Multiparous	71	46.9 ± 0.5	
Primiparous	51	33.3 ± 0.5	
Periparturient-period model for multiparous and primiparous cows			
Close-up strategy			0.44
HF	48	40.5 ± 0.6	
LF	91	39.9 ± 0.5	
Fresh strategy ^3^			0.52
LS	60	40.4 ± 0.6	
HS	79	39.9 ± 0.5	
Parity			<0.001
Multiparous	71	46.8 ± 0.4	
Primiparous	68	33.5 ± 0.5	

^1^ Far-off-period diet characterized as a controlled-energy (CE; <16.5% starch, ≥40% forage neutral detergent fiber (NDF)) or not-CE diet (NCE; ≥16.5% starch, <40% forage NDF or both). ^2^ Close-up-period diet characterized as a higher-forage NDF (HF; ≥40% forage NDF) or lower-forage NDF diet (LF; <40% forage NDF). ^3^ Fresh-period diet characterized as a lower-starch (LS; <25.5% starch) or higher-starch diet (HS; ≥25.5% starch).

**Table 8:** Least squares means ± SE of the herd average 305 d mature equivalent milk yield at the 4th test day (ME305) for the dry-period (*n* = 121 observations) and periparturient-period (*n* = 138 observations) nutritional strategies for multiparous and primiparous cows enrolled in a 72-farm prospective cohort study in the northeastern US.

Variable	*n*	ME305, kg	*p*-Value
Dry-period model for multiparous and primiparous cows			
Far-off strategy ^1^			0.90
CE	70	12,620 ± 177	
NCE	51	12,649 ± 200	
Close-up strategy ^2^			0.26
HF	41	12,799 ± 240	
LF	80	12,470 ± 172	
Periparturient-period model for multiparous and primiparous cows			
Close-up strategy			0.38
HF	47	12,767 ± 242	
LF	91	12,509 ± 176	
Fresh strategy ^3^			0.81
LS	59	12,603 ± 223	
HS	79	12,673 ± 198	

^1^ Far-off-period diet characterized as a controlled-energy (CE; <16.5% starch, ≥40% forage neutral detergent fiber (NDF)) or not CE-diet (NCE; ≥16.5% starch, <40% forage NDF or both). ^2^ Close-up-period diet characterized as a higher-forage NDF (HF; ≥40% forage NDF) or lower-forage NDF diet (LF; <40% forage NDF). ^3^ Fresh-period diet characterized as a lower-starch (LS; <25.5% starch) or higher-starch diet (HS; ≥25.5% starch).

**Table 9:** Least squares means ± SE of the 21-d pregnancy rate (PR) for the dry-period (*n* = 68 multiparous observations; *n* = 49 primiparous observations) and periparturient-period (*n* = 68 multiparous observations; *n* = 65 primiparous observations) nutritional strategies for multiparous and primiparous cows enrolled in a 72-farm prospective cohort study in the northeastern US. Herd 21-d PR was measured as the average of the two-21 d periods after the herd voluntary waiting period for the cows that calved during the same time frame as those sampled.

Variable	*n*	21 d PR	*p*-Value
Dry-period model for multiparous cows			
Far-off strategy ^1^			0.69
CE	39	23.2 ± 1.1	
NCE	29	23.8 ± 1.3	
Close-up strategy ^2^			0.14
HF	25	22.2 ± 1.4	
LF	43	24.7 ± 1.0	
Calving season ^3^			0.05
Warm	36	21.9 ± 1.1	
Cool	32	25.1 ± 1.2	
Dry-period model for primiparous cows			
Far-off strategy			0.95
CE	28	29.0 ± 1.5	
NCE	21	28.9 ± 2.0	
Close-up strategy			0.80
HF	17	29.3 ± 2.1	
LF	32	28.6 ± 1.4	
Far-off × close-up ^4^			0.07
CE × HF	12	31.7 ± 2.3 ^a^	
CE × LF	16	26.4 ± 2.0 ^b^	
NCE × HF	5	26.9 ± 3.5	
NCE × LF	16	30.8 ± 2.0 ^a^	
Periparturient-period model for multiparous cows			
Close-up strategy			0.14
HF	25	22.1 ± 1.3	
LF	43	24.7 ± 1.0	
Fresh strategy ^5^			0.75
LS	30	23.1 ± 1.2	
HS	38	23.7 ± 1.1	
Calving season			0.05
Warm	36	21.8 ± 1.1	
Cool	32	25.0 ± 1.2	
Periparturient-period model for primiparous cows			
Close-up strategy			0.49
HF	23	30.8 ± 1.7	
LF	42	29.3 ± 1.3	
Fresh strategy			0.22
LS	28	28.8 ± 1.5	
HS	37	31.3 ± 1.4	

^a–b^ Means with different superscripts differ based on unadjusted *p*-value comparisons (*p* < 0.20). ^1^ Far-off-period diet characterized as a controlled-energy (CE; <16.5% starch, ≥40% forage neutral detergent fiber (NDF)) or not-CE diet (NCE; ≥16.5% starch, <40% forage NDF or both). ^2^ Close-up-period diet characterized as a higher-forage NDF (HF; ≥40% forage NDF) or lower-forage NDF diet (LF; <40% forage NDF). ^3^ Calving season was dichotomized into warm (May through September) versus cool (October through April). ^4^ Means were compared with a Bonferroni test and comparisons to herds fed NCE during the far-off period and HF in the close-up period were not assessed due to a limited number of observations, as this is not a common nutritional strategy amongst farms in the northeastern United States. ^5^ Fresh-period diet characterized as a lower-starch (LS; <25.5% starch) or higher-starch diet (HS; ≥25.5% starch).

**Table 10:** Least squares means ± SE of the probability of pregnancy (PP) for the dry-period (*n* = 68 multiparous observations; *n* = 49 primiparous observations) and periparturient-period (*n* = 68 multiparous observations; *n* = 65 primiparous observations) nutritional strategies for multiparous and primiparous cows enrolled in a 72-farm prospective cohort study in the northeastern US. The herd PP was determined by averaging the PP for the first 2 estrus cycles after the herd voluntary waiting period for the group of cows that calved within the same calving date range as the cows observed using Dairy Comp 305 software calculations (the percent of services with confirmed pregnancy; [16]).

Variable	*n*	PP	*p*-Value
Dry-period model for multiparous cows			
Far-off strategy ^1^			0.90
CE	39	36.3 ± 1.4	
NCE	29	36.0 ± 1.7	
Close-up strategy ^2^			0.29
HF	25	34.9 ± 1.8	
LF	43	37.3 ± 1.4	
Calving season ^3^			0.01
Warm	36	33.2 ± 1.5	
Cool	32	39.0 ± 1.6	
Dry-period model for primiparous cows			
Far-off strategy			0.58
CE	28	42.7 ± 1.9	
NCE	21	44.3 ± 2.3	
Close-up strategy			0.17
HF	17	45.6 ± 2.5	
LF	32	41.3 ± 1.8	
Periparturient-period model for multiparous cows			
Close-up strategy			0.32
HF	25	34.9 ± 1.8	
LF	43	37.2 ± 1.4	
Fresh strategy ^4^			0.46
LS	30	35.3 ± 1.6	
HS	38	36.9 ± 1.5	
Calving season			0.009
Warm	36	33.2 ± 1.5	
Cool	32	38.9 ± 1.6	
Periparturient-period model for primiparous cows			
Close-up strategy			0.11
HF	23	45.4 ± 2.0	
LF	42	41.4 ± 1.5	
Fresh strategy			0.02
LS	28	40.4 ± 1.8	
HS	37	46.3 ± 1.6	
Close-up × fresh			0.14
HF × LS	11	40.6 ± 2.8 ^bY^	
HF × HS	12	50.1 ± 2.7 ^aX^	
LF × LS	17	40.2 ± 2.3 ^bY^	
LF × HS	25	42.5 ± 1.9 ^bY^	
Calving season			0.08
Warm	34	41.3 ± 1.6	
Cool	31	45.4 ± 1.7	

^a–b^ Means with different superscripts differ based on unadjusted *p*-value comparisons (*p* < 0.20). ^X–Y^ Means with different superscripts differ based on Tukey’s honest significance test and (*p* < 0.20). ^1^ Far-off-period diet characterized as a controlled-energy (CE; <16.5% starch, ≥40% forage neutral detergent fiber (NDF)) or not-CE diet (NCE; ≥16.5% starch, <40% forage NDF or both). ^2^ Close-up-period diet characterized as a higher-forage NDF (HF; ≥40% forage NDF) or lower-forage NDF diet (LF; <40% forage NDF). ^3^ Calving season was dichotomized into warm (May through September) versus cool (October through April). ^4^ Fresh-period diet characterized as a lower-starch (LS; <25.5% starch) or higher-starch diet (HS; ≥25.5% starch).

**Table 11:** Least squares means ± SE of the pregnancy risk to first service (PRFS) for the dry-period (*n* = 118 observations) and periparturient-period (*n* = 135 observations) nutritional strategies for multiparous and primiparous cows enrolled in a 72-farm prospective cohort study in the northeastern US.

Variable	*n*	PRFS	*p*-Value
Dry-period model for multiparous and primiparous cows			
Far-off strategy ^1^			0.94
CE	68	35.9 ± 2.3	
NCE	50	36.1 ± 2.7	
Close-up strategy ^2^			0.81
HF	42	36.4 ± 2.9	
LF	76	35.6 ± 2.1	
Parity			0.04
Multiparous	69	32.8 ± 2.2	
Primiparous	49	39.2 ± 2.6	
Calving season ^3^			0.006
Warm	61	31.0 ± 2.5	
Cool	57	41.0 ± 2.6	
Periparturient-period model for multiparous and primiparous cows			
Close-up strategy			0.91
HF	48	36.7 ± 2.7	
LF	87	36.3 ± 2.0	
Fresh strategy ^4^			0.45
LS	60	37.7 ± 2.5	
HS	75	35.2 ± 2.3	
Parity			0.01
Multiparous	69	32.8 ± 2.2	
Primiparous	66	40.2 ± 2.3	
Calving season			0.008
Warm	72	32.0 ± 2.3	
Cool	63	41.0 ± 2.5	

^1^ Far-off-period diet characterized as a controlled-energy (CE; <16.5% starch, ≥40% forage neutral detergent fiber (NDF)) or not-CE diet (NCE; ≥16.5% starch, <40% forage NDF or both). ^2^ Close-up-period diet characterized as a higher-forage NDF (HF; ≥40% forage NDF) or lower-forage NDF diet (LF; <40% forage NDF). ^3^ Calving season was dichotomized into warm (May through September) versus cool (October through April). ^4^ Fresh-period diet characterized as a lower-starch (LS; <25.5% starch) or higher-starch diet (HS; ≥25.5% starch).

## Data Availability

The data presented in this study is available upon reasonable request from the corresponding author.

## Supplementary Materials

The following supporting information can be downloaded at: https://www.mdpi.com/article/10.3390/ani13172701/s1, Table S1: The distribution of herds feeding a commercial anionic supplement during the close-up dry period, feeding monensin during the close-up and the fresh period, feeding rumen-protected choline during the close-up and fresh period, and routinely administering oral propylene glycol before or after parturition between nutrition strategies within each period for multiparous cows enrolled in a 72-farm prospective cohort study in the northeastern US.
