# Supplementary material for: Transition Cow Nutrition and Management Strategies of Dairy Herds in the Northeastern United States: Associations of Nutritional Strategies with Analytes, Health, Milk Yield, and Reproduction
Source: Animals (Basel). 2023 Aug 24;13(17):2701. doi: 10.3390/ani13172701 (PMC10486500; doi:10.3390/ani13172701)
Supplement: Supplementary file 1 [file animals-13-02701-s001.zip › animals-2564626-supplementary.pdf]

Table S1. The distribution of herds feeding a commercial anionic supplement during the close-up dry period, feeding monensin during the close-up and the fresh period, feeding rumen-protected choline during the close-up and fresh period, and routinely administering oral propylene glycol before or after parturition between nutrition strategies within each period for multiparous cows enrolled in a 72-farm prospective cohort study in the northeastern US.

Supplement and Period	Nutrition Strategy	n (%)	*P*-value
Anionic fed during close-up			
Far-off[1]	CE	33/43 (76.7%)	1.0
	NCE	23/29 (79.3%)	
Close-up[2]	HF	18/25 (72.0%)	0.39
	LF	38/47 (80.9%)	
Fresh[3]	LS	27/32 (84.4%)	0.27
	HS	29/40 (72.5%)	
Monensin fed during close-up			
Far-off	CE	39/43 (90.7%)	0.64
	NCE	28/29 (96.6%)	
Close-up	HF	22/25 (88.0%)	0.33
	LF	45/47 (95.7%)	
Fresh	LS	29/32 (90.6%)	0.65
	HS	28/40 (95.0%)	
Monensin fed during fresh			
Far-off	CE	40/43 (93.0%)	0.68
	NCE	26/29 (89.7%)	
Close-up	HF	22/25 (88.0%)	0.41
	LF	44/47 (93.6%)	
Fresh	LS	30/32 (93.8%)	0.69
	HS	36/40 (90.0%)	
RP choline fed during close-up			
Far-off	CE	13/43 (30.2%)	0.18
	NCE	7/29 (24.1%)	
Close-up	HF	7/25 (28.0%)	1.0
	LF	13/47 (27.7%)	
Fresh	LS	10/32 (31.3%)	0.60
	HS	10/40 (25.0%)	
RP choline fed during fresh			
Far-off	CE	8/43 (18.6%)	1.0
	NCE	5/29 (17.2%)	
Close-up	HF	4/25 (16.0%)	1.0
	LF	9/47 (19.2%)	
Fresh	LS	8/32 (25.0%)	0.22
	HS	5/40 (12.5%)	
Propylene glycol at parturition			
Far-off	CE	3/43 (7.0%)	0.64
	NCE	1/29 (3.5%)	

Close-up	HF	1/25 (4.0%)	1.0
	LF	3/47 (6.4%)	
Fresh	LS	0/32 (0%)	0.12
	HS	4/40 (10%)	

[1] Far-off-period diet characterized as a controlled-energy (CE; <16.5% starch, ≥40% forage neutral detergent fiber (NDF)) or not-CE diet (NCE; ≥16.5% starch, <40% forage NDF or both). [2] Close-up period diet characterized as a high-forage NDF (HF; ≥40% forage NDF) or low-forage NDF diet (LF; <40% forage NDF). [3] Fresh-period diet characterized as a low-starch (LS; <25.5% starch) or high-starch diet (HS; ≥25.5% starch).
